# Granzyme B in circulating CD8+ T cells as a biomarker of immunotherapy effectiveness and disability in neuromyelitis optica spectrum disorders

**DOI:** 10.3389/fimmu.2022.1027158

**Published:** 2022-11-09

**Authors:** Ziyan Shi, Qin Du, Xiaofei Wang, Jianchen Wang, Hongxi Chen, Yanling Lang, Lingyao Kong, Wenqin Luo, Mu Yang, Hongyu Zhou

**Affiliations:** ^1^ Department of Neurology, West China Hospital, Sichuan University, Chengdu, China; ^2^ Centre for Translational Research in Cancer, Sichuan Cancer Hospital & Institute, Chengdu, China; ^3^ School of Medicine, University of Electronic Science and Technology of China, Chengdu, China

**Keywords:** neuromyelitis optica spectrum disorders, NMOSD, granzyme B, GzmB, CD8, T cells, biomarker

## Abstract

**Background and objective:**

Neuromyelitis optica spectrum disorders (NMOSD) are chronical inflammatory demyelinating diseases of the central nervous system (CNS) and the underlying mechanism remains unclear. Several recent studies have demonstrated that T cells play a pivotal role in the pathogenesis of NMOSD.In this study, we investigated CD8+ T cell phenotypes and levels of the cytotoxic protein granzyme B (GzmB), as well as their potential clinical application in NMOSD.

**Methods:**

In this study, 90 peripheral blood samples were collected from 59 NMOSD patients with seropositive anti-aquaporin-4 (AQP4) antibodies and 31 sex- and age-matched healthy donors (HDs). Flow cytometry was used to detect circulating levels of GzmB and CD8+ T cell subpopulations, including naïve (T_N_, CCD7+CD45RA+), central memory (T_CM_, CCD7+CD45RA-), effector memory (T_EM_, CCD7-CD45RA-), terminal differentiation effector memory cells (T_EMRA_, CCD7-CD45RA+) in both groups. The associations between GzmB levels in CD8+T cells and clinical characteristics of NMOSD were evaluated.

**Results:**

NMOSD patients exhibited significantly decreased proportions of CD8+T_N_ cells and increased proportions of highly differentiated CD8+T cells (T_EMRA_) compared with HDs. In addition, levels of GzmB in CD8+ T cells were markedly higher in NMOSD patients than in HDs. Moreover, we observed that high proportions of GzmB-expressing CD8+ T cells were more common in patients with a poor response to immunotherapies, and showed a good potential to distinguish poor responders from responders (ACU=0.89). Clinical correlation analysis indicated that high levels of GzmB in CD8+ T cells were not only related to severe disability but also significantly associated with increased serum levels of neurofilament light (NFL) and glial fibrillary acidic protein (GFAP). Multivariate linear regression analyses further suggested that GzmB expression in CD8+ T cells was predominantly associated with disability and immunotherapy effectiveness in NMOSD, independent of the sex, age, and disease phase. Transcription factor T-bet in CD8+ T cells were also significantly elevated in NMOSD and were associated with increasing number of circulating CD8+T_EMRA_ cells and GzmB-expressing CD8+T cells.

**Conclusions:**

Our study support the involvement of GzmB-expressing CD8+ T cells in the inflammatory response in patients with NMOSD and provide a potential biomarker for disease immunotherapy effectiveness and disability progression.

## Introduction

Neuromyelitis optica spectrum disorders (NMOSDs) are inflammatory demyelinating diseases of the central nervous system (CNS) characterized by acute optic neuritis and transverse myelitis (1). Most cases are caused by serum pathogenic anti-aquaporin-4 (AQP4) antibodies that attack astrocytes of the optic nerve and spinal cord. Recurrence and disability rates are high in NMOSD patients, 90% of whom relapse within 3 years, and more than half of patients develop irreversible visual and/or motor disability within 5 years without treatment (2, 3). Numerous studies have shown that conventional immunosuppressants and immunomodulators can reduce the annual relapse rate and risk of disability in NMOSD patients; however, more than one third of patients still experience recurrent attacks and disability progression after immunotherapy (4, 5). Notably, three emerging therapies (eculizumab, satralizumab, and inebilizumab) have been demonstrated to be beneficial in preventing relapse of NMOSD in randomized controlled trials (6). Nevertheless, there is considerable heterogeneity in the response of different individuals to different therapies and a lack of biomarkers for predicting the efficacy of NMOSD drugs. Thus, there is an urgent need to explore the mechanisms underlying the NMOSD and identify new therapeutic targets and biomarkers for predicting disease progression.

T cells play a crucial role in the pathogenesis of NMOSD. Serum autoantibodies against the water channel protein aquaporin-4 (AQP4) are T-cell-dependent antibodies in most NMOSD patients. Autoreactive T cells have been observed in the peripheral blood and within the inflammatory CNS lesions (7–9). Furthermore, animal models have suggested that autoreactive T cells are sufficient to induce NMO-like lesions in the absence of anti-AQP4 antibodies (10), indicating that autoreactive T cells play a pivotal role in NMOSD pathogenesis. Thus, understanding the role of T cells and related cytokines in NMOSD may aid in identifying potential treatment targets and novel biomarkers for predicting disease progression. Previous studies of T cells in NMOSD have mainly focused on CD4+ T cells, while the key role of CD8+ T cells has been largely overlooked. Increasing evidence suggests that cytotoxic CD8+ T cells play an important role in CNS autoimmune diseases including multiple sclerosis (MS), Susac syndrome, and NMOSD (11–13).

In addition to secreting pro-inflammatory cytokines that promote CNS inflammation, cytotoxic CD8+ T cells can also induce target cell death by releasing serine protease granzyme B (GzmB). Previous studies have indicated that cytotoxic CD8+ T cell infiltration in the brain lesions of MS patients mediates axonal injury and neuronal death, and that inhibition of this damage induces neuroprotection *in vitro* and *in vivo* (14, 15). Similarly, researchers have observed that suppression of GzmB expression can effectively reduce inflammatory lesions and disease severity in patients with autoimmune skin diseases (16, 17). These findings support the notion that cytotoxic CD8+T cells expressing GzmB promote autoimmune and neurological processes, highlighting their potential as a therapeutic target and biomarker of disease progression. However, the regulatory mechanisms underlying phenotypic differentiation and functional expression of CD8+ T cells remain unclear.

T-bet is a key transcription factor that regulates CD8+ T-cell activation and differentiation (18). In chronic infection, T-bet promotes CD8+ T cell transfer from central memory (T_CM_) to effector memory (T_EM_) and terminally differentiated effector memory (T_EMRA_) cells and sustains virus-specific CD8+ T cell responses, including increases in the expression of cell lysis-related protein (GzmB) and the pro-inflammatory cytokines interferon γ (IFNγ) and tumor necrosis factor α (TNFα) (19, 20). Although the regulation of CD8+ T phenotypic differentiation and functional expression by T-bet has been studied in the field of viral infection and cancers, knowledge of its regulatory role in NMOSD remains unknown (21, 22).

Previously, we observed increasing proportions of activated CD8+ T cells in the peripheral blood of NMOSD patients, along with elevated levels of INFγ and TNFα, indicating that CD8+ T cells may be involved in the peripheral inflammation associated with NMOSD (13). In this study, we further investigated the potential clinical value of CD8+ T cell phenotypes and levels of GzmB in NMOSD patients. Our findings indicated that the proportions of CD8+ T_EMRA_ cells in the peripheral blood were significantly increased in NMOSD patients who also exhibited high levels of the cytotoxic protein GzmB. Notably, these increased proportions of GzmB+CD8+ T cells were significantly associated with Expanded Disability Status Scale (EDSS) scores, serum neurofilament light (NFL), serum glial fibrillary acidic protein (GFAP) levels, as well as immunotherapy efficacy in NMOSD patients. In conclusion, our findings suggest GzmB+CD8+ T cells as a potential biomarker treatment effectiveness and disability in NMOSD patients.

## Materials and methods

### Participants

This study was approved by the Medical Ethics Committee of the West China Hospital, Sichuan University and all participants given informed consent prior to their inclusion in this study. Patients diagnosed with NMOSD were recruited from the West China Hospital of Sichuan University between January 2021 and December 2021 (n=59). All patients met the 2015 diagnostic criteria and were seropositive for anti-AQP4 antibodies (EUROIMMUN AG, Luebeck, Germany) (23). Sex- and age-matched healthy donors (HDs) were also enrolled (n=31). EDSS scores were used to assess disability status in each patient with NMOSD (24). The acute phase was defined as within 2 months of onset, while the remission phase was defined as greater than 2 months after attack. Detailed clinical characteristics are summarized in **Table 1**, including age, sex, disease phases, EDSS scores, and treatments. NMOSD patients were divided into a response group and a poor response group according to their response to immunosuppressive or immunomodulatory therapies (IMT) during remission (treatments lasting for at least 12 months). A response to IMT was defined as relapse free or only 1 mild relapse during treatment, while a poor response to IMT was defined as ≥ 2 relapses or 1 severe relapse (25).

**Table 1:** Demographic and clinical characteristics of participants.

	HD (n=31)	NMOSD (n=59)	P values
Gender, Female (%)	26 (84%)	52 (90%)	0.745
Age, mean ± SD, years	42.6 ± 13.29	45.87 ± 14.53	0.250
EDSS scores, median (range)		4 (0-8.5)	
Disease phases, n			
Relapse (≤2 months)		27	
Remission (>2months)		32	
Treatments, n			
Untreated		12	
Steroids		8	
IMT (AZA/MMF/RTX)^a^		39 (4/27/8)	
Responders (AZA/MMF/RTX)		14 (2/11/1)	
Poor responders (AZA/MMF/RTX)		12 (0/7/5)	

HD, healthy donors; NMOSD, neuromyelitis optica spectrum disorders; EDSS, Expanded Disability Status Scale; IMT, immunosuppressive or immunomodulatory therapies; AZA, azacytidine, 2mg/kg per day; MMF, mycophenolate mofetil, 20mg/kg per day; RTX, rituximab, 500-1000mg per 6 months. aExclude 8 NMOSD patients with treatment less than 1 year, and 5 NMOSD patients with irregular treatment.

### Flow cytometry

Peripheral blood samples from patients with NMOSD and HDs were prepared as single-cell suspensions and stained for flow cytometry, as previously described (13). Single-cell suspensions were stained with the selected antibodies for 30 min after incubation with Human TruStain FcX™ (BioLegend) at 4°C. Anti-human CD3-APC (OKT3), CD8a-PerCP (RPA-T8), CD45RA-PE (HI100), and CCR7-APC/Cyanine7 (G043H7) (BioLegend) were used to label the surface markers of the CD8+ T cells. Granzyme B-FITC (QA16A02) and Tbet-PE/Cyanine7 (4B10) (BioLegend) were used for intracellular staining after fixation and permeabilization with the Foxp3/Transcription factor staining buffer (Invitrogen). Samples were acquired using an FACS Canto II flow cytometer (BD Biosciences), and the original data analysis was performed using FlowJo v10 (BD Biosciences).

### Neurofilament light (NFL) and GFAP detection

Serum samples were centrifuged at 2,000 *g* for 10 min at room temperature and stored at -80°C for later use. Serum levels of NFL and GFAP were measured in patients with NMOSD (n=18) using a commercial Simoa Human Neurology 2-Plex B assay (N2PB) kit in accordance with the manufacturer’s protocol (serum 1:4 dilution) (26).

### Statistical analysis

Statistical analyses were performed using GraphPad Prism V8.0 (GraphPad Software, San Diego, California, USA) and/or SPSS software V25.0 (IBM Corp., Armonk, NY, USA). Continuous variables are described as mean with standard deviation (SD) and/or median with range. Categorical variables are presented as numbers and percentages. The Mann–Whitney U-test was used to compare continuous variables between two groups, while the Kruskal–Wallis test with Dunn’s test for multiple comparisons was used for comparisons among three or more groups. The chi-square test was used to compare categorical variables between groups. Comparisons of CD8+ T cell subpopulations and cytotoxic function between the groups were adjusted for age and sex using linear regression models. Receiver operating characteristic (ROC) curves were performed to evaluate the accuracy of the GzmB-expressing CD8+T cell subsets in distinguishing immunotherapies responders from poor responders. Spearman correlation analysis was used to evaluate the correlation of GzmB levels in CD8+ T cell subsets with EDSS score, serum NFL, and GFAP. Multivariate linear regression analysis was used to estimate the correlation between levels of GzmB in CD8+ T cell subsets and clinical characteristics, including sex, age, EDSS scores, disease phase, and treatments. The level of statistical significance was defined as a two-tailed p value < 0.05.

## Results

### Demographic and clinical characteristics of participants

The demographic and clinical characteristics of the participants are summarized in **Table 1**. A total of 90 peripheral blood samples were collected from 59 patients with NMOSD and 31 HDs. There were no significant differences in sex or age between the NMOSD and HD groups. During sample collection, 12 patients with NMOSD were admitted without any immunotherapy, eight patients were treated with glucocorticoids (GC), and 39 patients were treated with IMT (azacytidine [AZA], n=4; mycophenolate mofetil [MMF], n=27; and rituximab [RTX], n=8). Among the patients who received IMT, 26 received treatment for more than 1 year. These patients were further divided into responder (n=14) and poor responder (n=12) according to their response to IMT.

### Circulating CD8+T cells exhibit high differentiation and increased GzmB expression in patients with NMOSD

**Figure 1:**
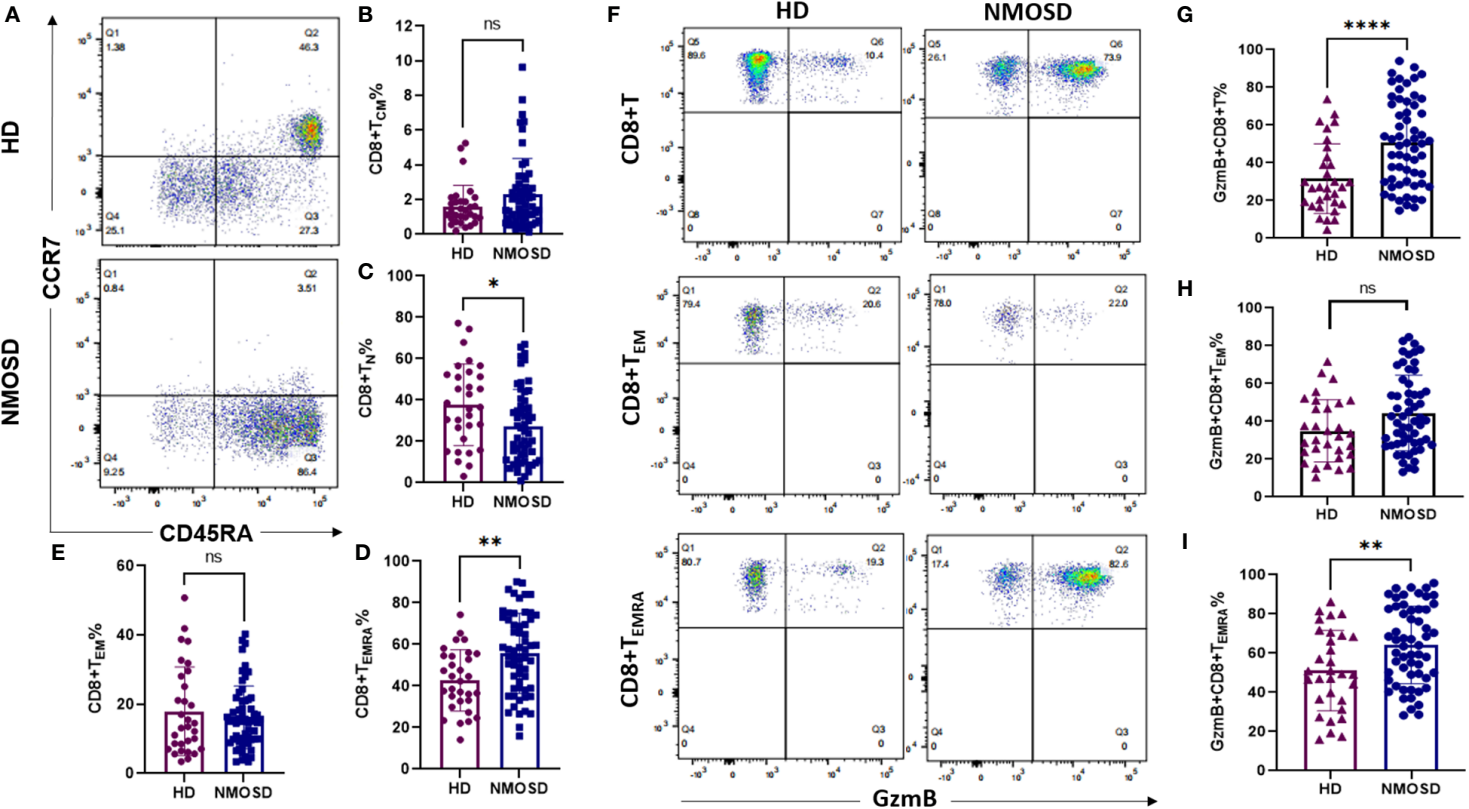
Circulating CD8+ T cell subpopulations and GzmB expression in patients with NMOSD and HD. Peripheral blood was collected from healthy donors **(HD)** (n=31) and patients with neuromyelitis optica spectrum disorder (NMOSD) (n=59). **(A)** The flow cytometric gating strategy for CD8+ T cell subpopulations. Central memory (T_CM_, CCD7+CD45RA-), naïve (T_N_, CCD7+CD45RA+), effector memory (T_EM_, CCD7-CD45RA-), and terminal differentiation effector memory T cells (T_EMRA_, CCD7-CD45RA+). **(B–E)** Comparison of CD8+ T cell subsets between HD and NMOSD. **(F–I)** Proportions of GzmB-expressing CD8+ T, CD8+ T_EM_, and CD8+ T_EMRA_ cells were further measured by flow cytometry. ****P<0.0001, **P < 0.01, *P < 0.05 and ns (not significant) by Mann–Whitney U-test.

We compared the memory and/or effector characteristics of peripheral CD8+ T cells in the NMOSD and HD groups based on the expression of CCR7,CD45RA, and GzmB (27) (**Figure 1**). Based on the gating strategy of flow cytometry, CD8+ T cells were divided into four subgroups: naïve (T_N_, CCR7+CD45RA+), central memory (T_CM_, CCR7+CD45RA-), effector memory (T_EM_, CCR7-CD45RA-), and terminally differentiated effector memory (T_EMRA_, CCR7-CD45RA+) cells (**Figure 1A**). Our results revealed that patients with NMOSD had significantly lower proportions of CD8+ T_N_ cells than HDs (median, 24.9% vs. 36.5%, P=0.0185) (**Figure 1C**). In contrast, the highly differentiated CD8+ T cell subpopulation (CD8+ T_EMRA_) was more strongly expressed in patients with NMOSD than in HDs (median: 56.9% vs. 42.3%, P=0.0019) (**Figure 1D**). However, the subpopulations of T_CM_ and T_EM_ were comparable between patients with NMOSD and HDs (**Figures 1B, E**). These findings indicate that highly differentiated CD8+ T cells are more common in the blood of patients with NMOSD than in that of HDs.

To explore the cytotoxic function of CD8+ T cells in each group, we further investigated the expression of the cytolytic protein GzmB in CD8+T cells and their effector subsets (T_EM_ and T_EMRA_) (**Figure 1F**). Compared with HDs, patients with NMOSD exhibited significantly increased proportions of GzmB+CD8+ T cells (50.2% vs. 27.1%, P<0.0001) and GzmB+CD8+ T_EMRA_ cells (64% vs. 49.4%, P=0.0062) (**Figures 1G, I**). Nevertheless, levels of GzmB+CD8+ T_EM_ cells did not significantly differ between the NMOSD and HD groups (**Figure 1H**). Our data further demonstrated that CD8+ T cells in the blood of patients with NMOSD are not only highly differentiated but also express a large amount of GzmB, suggesting that CD8+ T cells are involved in the peripheral inflammation associated with NMOSD.

### GzmB-expressing CD8+ T cells as potential biomarkers for predicting the effectiveness of immunotherapies in patients with NMOSD

**Figure 2:**
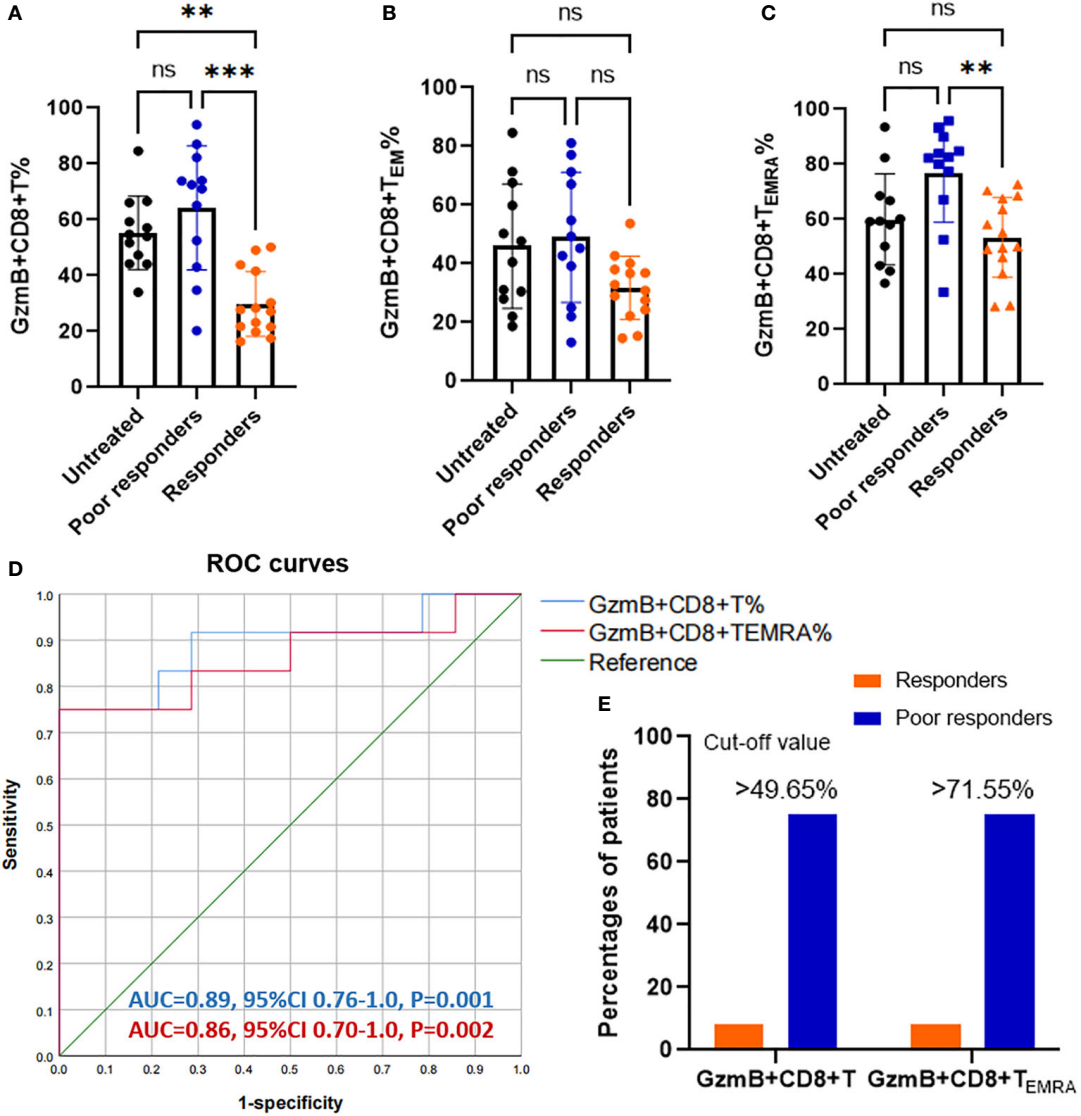
Increasing proportions of GzmB-expressing CD8+ T cells in poor responders with NMOSD. **(A–C)** Patients with neuromyelitis optica spectrum disorder (NMOSD) were divided into three groups according to the response to immunotherapies, including untreated (NT) patients (n=12), responders (n=14), and poor responders (n=12). Poor responders exhibited a significant increase in GzmB+CD8+ T% **(A)** and GzmB+CD8+ T_EMRA_% **(C)**, but not in GzmB+CD8+ T_EM_% **(B)**. **(D)** Receiver operating characteristic (ROC) curve for evaluating the diagnostic sensitivity and specificity of GzmB-expressing CD8+ T cells in responders and poor responders. Diagnostic accuracy was assessed based on the area under the curve (AUC). **(E)** Percentages of patients with levels of GzmB-expressing CD8+ T cells over the cut-off value in responders and poor responders. GzmB: granzyme B **P<0.01, ***P<0.001, and ns (not significant) by Kruskal-Wallis test with Dunn’s multiple comparisons test.

To assess the effect of immunotherapies on levels of GzmB expression in CD8+ T cells, we divided patients with NMOSD into untreated patients (n=12), responders to immunotherapies (n=14), and poor responders to immunotherapies (n=12). The proportions of GzmB+CD8+ T cells in untreated patients and poor responders were comparable, and both were significantly higher than those in responders (P=0.0033 and P=0.0002, respectively) (**Figure 2A**). Similarly, proportions of GzmB+CD8+ T_EMRA_ cells were also significantly higher in poor responders than in responders, but not those in untreated patients (**Figure 2C**). No significant differences in the proportions of GzmB+CD8+ T_EM_ cells were observed among the three groups (**Figure 2B**). In addition, we further compared the proportions of circulating GzmB-expressing CD8+T cells between responders to MMF (n=11) and poor responders to MMF (n=7), and showed that proportions of GzmB+ CD8+T cells were significantly higher in poor responders (P=0.034) (**Supplementary Figure 1A**). GzmB+CD8+T_EM_ and GzmB+CD8+T_EMRA_ showed the similar trends, although there was no statistically significant difference (**Supplementary** **Figures 1B, C**).

Given that GzmB levels in CD8+ T cells significantly differed between patients with good and poor responses to immunotherapies, we next performed a receiver operating characteristic (ROC) curve analysis to assess the percentage of GzmB+CD8+ T cells as a potential biomarker for differentiating the response to immunotherapies. As expected, the percentage of GzmB+CD8+ T cells exhibited good diagnostic potential in distinguishing poor responders from responders (area under the curve [AUC]=0.89, P=0.001), whereas GZMB+CD8+ T_EMRA_ had a relatively lower AUC of 0.86 (P=0.002) (**Figure 2D**). Subsequently, ROC curve analysis further indicated that the cut-off values for %GzmB+CD8+ T cells and %GZMB+CD8+ T_EMRA_ cells were 49.65% and 71.55%, respectively, and that 75% of the patients above the cut-off value exhibited poor responses to immunotherapies (**Figure 2E**). Our data suggest that high levels of GzmB in CD8+ T cells are associated with a poor response to immunotherapies in patients with NMOSD, highlighting their potential as a biomarker for predicting the effectiveness of immunotherapies.

### High proportions of GzmB-expressing CD8+ T cells are associated with disability in patients with NMOSD

**Figure 3:**
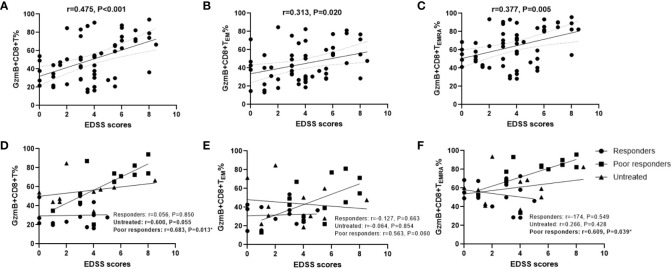
High proportions of GzmB-expressing CD8+ T cells are associated with disability in patients with NMOSD. **(A–C)** Correlation between Expanded Disability Status Scale (EDSS) scores and the percentages of GzmB+CD8+ T cells **(A)**, GzmB+CD8+ T_EM_ cells **(B)**, and GzmB+CD8+ T_EMRA_ cells **(C)** (Spearman correlation analysis). **(D–F)** Correlation analysis between EDSS scores and GzmB-expressing CD8+ T cell subpopulations according to the different immunotherapies and response, including responders group (n=14), untreated group (n=12), and poor responders (n=12) (Spearman correlation analysis). GzmB: granzyme **(B)** ns (not significant) by Mann–Whitney U-test. * indicates statistical significance (P<0.05).

Because GzmB levels in peripheral CD8+ T cells were significantly increased in patients with NMOSD, we explored whether high levels of GzmB play an active role in the disease process. First, we examined the relationship between disability (EDSS scores) and the proportions of GzmB+CD8+ T cell subsets in the NMOSD group (n=59). Spearman correlation analysis indicated that EDSS scores were moderately correlated with the proportions of GzmB+CD8+ T cells in patients with NMOSD (r=0.476, P<0.001)(**Figure 3A**), and similar relationships were also observed in GzmB+CD8+ T_EM_ and GzmB+CD8+ T_EMRA_ subpopulations (**Figures 3B, C**). Subsequently, we further explored the correlation between GzmB expressions of CD8+T cells and disability under different treatment conditions (**Figures 3D–F**). As results, we found a significant association between proportions of GzmB+CD8+ T cells and EDSS scores mainly in the poor-response group (r=0.682, P=0.013), and the same trend was observed in untreated patients, although the difference did not reach statistical significance (r=0.600, P=0.055) (**Figure 3D**). However, we did not find this association in response group, suggesting that effective immunotherapy may inhibit GzmB expression in CD8+T cells, thereby eliminating the correlation between GzmB levels and disability in NMOSD.

Next, we evaluated the association between the clinical characteristics of NMOSD and GzmB expression in circulating CD8+ T cells and their effector subsets (T_EM_ and T_EMRA_) using multivariate linear regression models. In the NMOSD cohort, the proportion of GzmB+CD8+ T cells was independently associated with EDSS scores (β=4.47, P=0.005) and treatments (β=-10.01, P=0.010) (**Table 2**). However, no significant correlations were observed between GzmB+CD8+ T cells and sex, age, or disease stage. In the analysis of GzmB expression levels in effector subsets of CD8+ T cells, we found that the proportion of Gzmb+CD8+ T_EMRA_ cells exhibited a trend of positive correlation with EDSS scores (β=3.23, P=0.056) (**Table 2**). These results indicate that levels of GzmB expression in CD8+ T cells are not only closely related to disability but are also influenced by immunotherapie.

**Table 2:** Associations between GzmB levels in CD8+ T subpopulations and clinical characteristics of NMOSD patients.

	GzmB+CD8+T				GzmB+CD8+T_EM_			GzmB+CD8+T_EMRA_		
	β	95% CI	P values		β	95% CI	P values	β	95% CI	P values
Sex										
Male	Reference									
Female	-8.21	-22.38,5.96	0.246		0.10	-16.70,16.90	0.991	-7.74	-23.34,7.85	0.319
Age, years	0.18	-0.30,0.66	0.443		-0.07	-0.64,0.50	0.812	0.18	-0.35,0.71	0.486
Disease phases										
Remission	Reference									
Relapse	-5.88	-18.62,6.85	0.354		2,72	-12.37,17.82	0.716	-3.67	-17.68,10.34	0.597
EDSS scores	4.47	1.46,7.48	**0.005**		2.25	-0.1.32,5.81	0.209	3.23	-0.08, 6.54	0.056
Treatments 1=Untreated, 2=Poor responders, 3=Responders	-10.01	-17.40,-2.62	**0.010**		-5.14	-13.90,3.62	0.240	-0.83	-8.96,7.30	0.836

NMOSD, neuromyelitis optica spectrum disorders; EDSS, Expanded Disability Status Scale. The F values and P-values of multivariate linear regression models for GzmB+CD8+T (F=7.26, P<0.001), GzmB+CD8+TEM (F=1.183, P=0.340), and GzmB+CD8+TEMRA (F=2.111, P=0.091). The bold indicates statistical significance (P<0.05).

**Figure 4:**
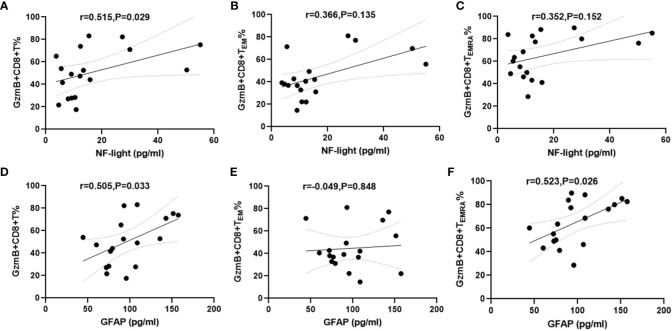
Positive correlation between proportions of GzmB+CD8+ T cells and serum NFL and GFAP levels in patients with NMOSD. **(A–C)** Association between the proportion of GzmB-expressing CD8+ T cells and serum NFL (n=18). **(D–F)** Association between the proportion of GzmB-expressing CD8+ T cells and serum GFAP (n=18) by spearman correlation analysis. GzmB, granzyme B; NMOSD, neuromyelitis optica spectrum disorder; NFL, neurofilament light; GFAP, glial fibrillary acidic protein.

Increased serum NFL and GFAP concentrations are correlated with CNS damage and have been regarded as biomarkers of disease activity and disability in patients with NMOSD (26). Therefore, serum samples and PBMC were simultaneously collected from 18 patients with NMOSD to evaluate the relationship between GzmB expression in CD8+ T cells and serum levels of NFL and GFAP. The mean concentrations and standard deviation (SD) of serum NFL and GFAP were 16.6 ± 14.87 pg/mL and 97.78 ± 31.83 pg/mL, respectively. Correlation analyses revealed that serum NFL levels were positively associated with %GzmB+CD8+ T cells (r=0.515, P=0.029) (**Figure 4A**), but not with %GzmB+CD8+ T_EM_ cells or %GzmB+CD8+ T_EMRA_ cells (**Figures 4B, C**). Moreover, serum GFAP levels were positively correlated not only with %GzmB+CD8+ T cells (r=0.505, P=0.033), but also with %GzmB+CD8+ T_EMRA_ cells (r=0.523, P=0.026), although there was no significant correlation with %GzmB+CD8+ T_EM_ cells (**Figures 4D–F**). These findings further support our hypothesis that high GzmB expression in CD8+ T cells is closely related to disease progression and may be used as a peripheral biomarker of CNS injury in NMOSD.

### Up-regulation of T-bet is associated with increased levels of GzmB in circulating CD8+ T cells

**Figure 5:**
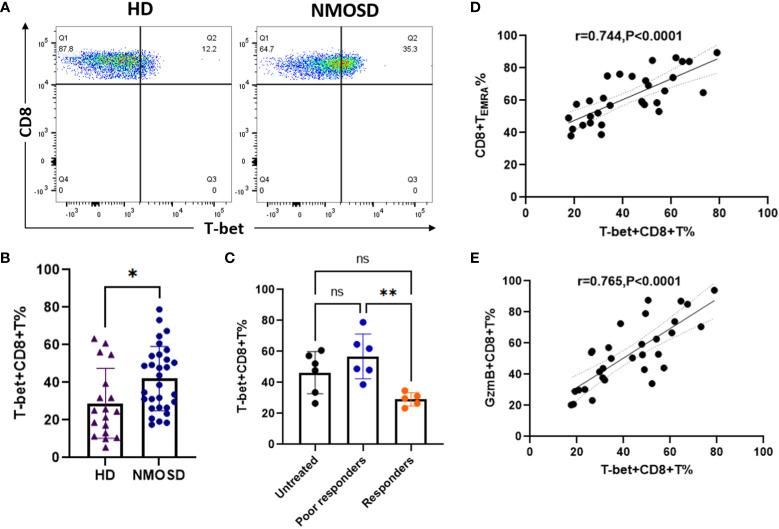
Upregulation of the transcription factor T-bet is associated with high expression of GzmB in circulating CD8+ T cells. **(A)** The flow cytometric gating strategy for evaluate levels of T-bet in CD8+ T cells. **(B)** Comparison of T-bet+CD8+ T cells between patients with NMOSD (n=31) and HDs (n=6) (Mann–Whitney U-test). **(C)** Comparison of T-bet+CD8+ T cells among untreated patients (n=6), poor responders (n=6), and responders (n=5) (one-way ANOVA with Tukey’s multiple comparison test). **(D,E)** The correlation between T-bet expression and the proportions of CD8+ T_EMRA_ cells **(D)** or GzmB+CD8+ T cells **(E)** in patients with NMOSD. GzmB, granzyme B; NMOSD, neuromyelitis optica spectrum disorder. *P<0.05, **P<0.01, and ns (not significant).

Transcription factor T-bet plays an important role in the activation and functional expression of CD8+ T cells. In this study, we investigated whether high GzmB expression in CD8+ T cells is associated with the un-regulation of the transcription factor T-bet in patients with NMOSD. Flow cytometry analysis was performed to detect the expression of T-bet in CD8+ T cells in patients with NMOSD and HDs (**Figure 5A**). Compared with HDs, patients with NMOSD exhibited a significant increase in the percentage of T-bet+CD8+ T cells (24.8% vs. 41.25%, P=0.015) (**Figure 5B**). Furthermore, patients who responded to immunotherapies exhibited significantly lower percentages of T-bet+CD8+ T cells than poor responders (29.12% vs. 56.85%, P=0.005), while no significant difference was observed between untreated patients and poor responders (**Figure 5C**). Further correlation analysis revealed that T-bet+CD8+ T cell were significantly related to highly differentiated CD8+T cell subpopulations (T_EMRA_) (r=0.744, P<0.0001) (**Figure 5D**) and GzmB+CD8+ T cells (r=0.765, P<0.0001) (**Figure 5E**).

These findings suggest that levels of T-bet+CD8+ T cells are increased in the peripheral blood in patients with NMOSD and are associated with hyper differentiation and enhanced cytotoxic function of CD8+ T cells. Thus, inhibiting CD8+ T cell activation and cytotoxicity may reduce peripheral inflammatory responses in patients with NMOSD.

## Discussion

It is widely accepted that autoreactive T cells are activated in the periphery and migrate to the CNS through the blood–brain barrier (BBB) to participate in CNS inflammatory demyelination (28). Although previous studies on CNS autoimmune diseases have mainly focused on CD4+ T cells, histopathological findings from autopsy and biopsy studies suggest that CD8+ T cells are the predominant immune cells infiltrating CNS lesions and exhibit tissue-resident memory and cytotoxicity (29, 30). Our previous study found that circulating CD8+ T cells were abnormally activated in patients with NMOSD and expressed high levels of the pro-inflammatory cytokines IFNγ and TNFα, which may be involved in peripheral inflammatory responses and promote disease progression (13). In the current study, we further demonstrated that CD8+ T cells in the peripheral blood of patients with NMOSD exhibited a highly differentiated phenotype (T_EMRA_) and expressed considerable levels of the cytolytic protein GzmB. Furthermore, the increasing numbers of GzmB-expressing CD8+ T cells were associated with a poor response to immunotherapies and severe disability in the NMOSD group. Levels of the transcription factor T-bet in circulating CD8+ T cells were also significantly elevated in patients with NMOSD and contributed to increasing of terminal differentiation of CD8+ T cells and high expression of GzmB. Our data provide insight into a novel biomarker for predicting the effectiveness of immunotherapies and disability in patients with NMOSD, as well as a potential target for treatments.

Although T cells are considered to be involved in the pathogenesis and development of NMOSD, the key role of CD8+ T cell phenotypes and function in disease progression remains poorly understood. In this study, we observed significantly elevated proportions of CD8+ T_EMRA_ cells and significantly enhanced expression of the neurotoxic mediator GzmB in the peripheral blood of patients with NMOSD. Increasing evidence suggests that cytotoxic CD8+ T cells are involved in the development of multiple autoimmune diseases. Blanco et al. reported a significant increase in CD8+ T_EMRA_ cells expressing high levels of GzmB in the peripheral blood of patients with systemic lupus erythematosus (SLE), which was significantly associated with the clinical activity of the disease (31). In addition, abnormal amplification of cytotoxic CD8+ T cells has been observed in the peripheral blood and brain lesions of patients with Susac syndrome, in whom secreted GzmB adhered to CNS microvessels in different lesion regions, resulting in vascular endothelial cell injury, BBB disruption, and microbleeding (12). Intervention with GzmB significantly improved disease progression in the mouse model of Susac syndrome. Furthermore, Fransen et al. found that increased clustering of CD8+ T cells in the perivascular space correlated with inflammatory lesion activity and demyelinated lesion load in patients with chronic progressive MS (29). These studies imply that activated CD8+ T cells in the peripheral blood may enter the CNS by disrupting the BBB and participating in neuroinflammatory responses and lesion activity. Additionally, evidence from EAE models demonstrates that treatment with a GzmB inhibitor (Serpina3n) can effectively reduce cytotoxic CD8+T cell-mediated axonal and neuronal damage in the CNS, as well as T-cell-mediated neuropathic pain (15). These findings strongly suggest that cytotoxic CD8+ T cells promote inflammatory responses and disease progression in a variety of autoimmune and neurological diseases by secreting GzmB, making GzmB a potential therapeutic target for these diseases and NMOSD.

In addition to GzmB-expressing CD8+T cells, the role of Gzmb-producing B cells has also been widely noticed in autoimmune diseases. GzmB-producing B cells are considered a type of regulatory B cells that have been found to be involved in the pathogenesis of multiple autoimmune diseases. Serval studies have revealed that GzmB-producing regulatory B cells are decreased in peripheral blood of systemic lupus erythematosus (SLE), lupus nephritis, and rheumatoid arthritis (RA), which are closely associated with poor prognosis of these diseases (32–34). On the other hand, a recent study found that circulating Gzmb+CD8+T cells and Gzmb+CD19+ B cells were significantly increased in MS patients during fingolimod and natalizumab treatments (35), suggesting that B cells may exhibit cytotoxic behavior similar to CD8^+^ T lymphocytes in MS patients under different treatments. These results imply that circulating GzmB-expressing B cells and GzmB-expressing CD8+T cells seem to play different or even opposite roles in the pathogenesis of autoimmune diseases, but whether they interact and how the mechanism is still unknown in NMOSD. We believe that further study of circulating GzmB-releasing from CD8+T cells and B cells in NMOSD is warranted and interesting, which will help us to have a deeper understanding of the mechanism of T cell-B cell interaction in this disease.

Immunotherapies, including AZA, MMF, and RTX, are currently the most used agents for preventing relapse and disability in patients with NMOSD. However, studies have shown that more than 30% of patients still experience frequent relapse and disability progression under the first-line immunotherapies, and there are no biomarkers that can reflect the effectiveness of immunotherapies (4, 5). In this study, we discovered that a high proportion of GzmB-expressing CD8+ T cells was significantly associated with a poor response to immunotherapies and showed good potential for predicting the efficacy of immunotherapies (AUC=0.89). Moreover, our study provides a cut-off value of %GzmB+CD8+ T cells for distinguishing patients who are sensitive to immunotherapies. Based on this cutoff value, 75% of poor responders and only 8% of responders were classified as having high GzmB status. Our study not only confirms that GzmB+CD8+ T cells are involved in NMOSD, but also provides insight into a novel biomarker for predicting the efficacy of first-line immunotherapies, highlighting the need for validation in longitudinal cohort studies. In the future study, it remains necessary to evaluate the role of GzmB-expressing CD8+ T cells in monitoring response to different immunotherapies in NMOSD.

Additionally, our correlation analysis indicated that elevated levels of GzmB in CD8+ T cells were markedly associated with severe disability in patients with NMOSD, independent of sex, age, or disease phase. The significant association between GzmB levels in CD8+ T cells and EDSS scores suggests that GzmB levels in CD8+ T cells are potential biomarkers of disability in NMOSD. Serum levels of NFL and GFAP are widely considered biomarkers of neuroaxonal damage and astrocyte injury, respectively, both of which can reflect disease activity and disability in NMOSD (26). Notably, our study revealed that GzmB levels in CD8+ T cells were positively correlated with serum concentrations of NFL and GFAP, indicating that GzmB expression on peripheral CD8+ T cells may reflect CNS injury in patients with NMOSD. These results support our hypothesis that cytotoxic CD8+ T cells mediate CNS injury by secreting the neurotoxic mediator GzmB and are involved in peripheral and central inflammatory responses in patients with NMOSD.

The differentiation and functional expression of CD8+ T cells are affected by the transcription factor T-bet in the context of chronic infectious disease. Therefore, we further investigated the relationship between T-bet and peripheral CD8+ T_EMRA_ and GzmB+CD8+ T cells in the NMOSD group. Our data revealed that the proportion of T-bet+CD8+ T cells in the peripheral blood was significantly higher in patients with NMOSD than in HDs, whereas it was significantly decreased in patients with a good response to immunotherapies. These findings indicate that levels of T-bet are elevated in the peripheral blood of patients with NMOSD and are regulated by immunotherapy drugs. Importantly, our data further demonstrated that those with high levels of T-bet in CD8+ T cells displayed an increasing proportion of CD8+ T_EMRA_ cells and GzmB expression. In chronic viral infection, T-bet represses the expression of programmed cell death protein 1 (PD-1) and other related inhibitory receptors and sustains functional CD8+ T cell responses (19). Evidence from epigenetic studies and chromatin mapping of human CD8+ T cells *via* ATACT-seq has verified that levels of T-bet and proximity effector genes (Gzmb and Prf1) are upregulated in circulating CD8+ T_EMRA_ cells (27). These data are consistent with our findings, suggesting that T-bet enhances the differentiation and cytotoxic function of CD8+ T cells and promotes disease progression and deterioration in inflammatory CNS diseases such as NMOSD. Taken together, our results suggest that increases in the proportion of CD8+ T_EMRA_ cells and the related effector molecules T-bet and GzmB are associated with poor outcomes in patients with NMOSD. Thus, T-bet and GzmB represent potential therapeutic targets for NMOSD.

Our study was limited by the cross-sectional design and lack of longitudinal follow-up information. Therefore, a prospective cohort study is required to confirm the prognostic predictive value of cytotoxic CD8+ T cells in patients with NMOSD. In addition, patients receiving immunotherapies were not excluded when detecting serum concentrations of NFL and GFAP in this study, thus the association with circulating GzmB-expressing CD8+T cells might be affected. Therefore, it is necessary to include more untreated patients in the future study to clarify the relationship between GzmB-expressing CD8+T cells and serum NFL and GFAP. Nonetheless, our findings support the involvement of cytotoxic CD8+ T cells in the inflammatory response to NMOSD and provide insight into a potential biomarker for the effectiveness of immunotherapies and disability progression in NMOSD.

## Data availability statement

The original contributions presented in the study are included in the article/**Supplementary Material**. Further inquiries can be directed to the corresponding authors.

## Ethics statement

The studies involving human participants were reviewed and approved by Medical Ethics Committee of the West China Hospital, Sichuan University. The patients/participants provided their written informed consent to participate in this study.

## Author contributions

Study conception and design: ZS, MY, and HZ. Collection of samples: QD, JW, XW, HC, YL, LYK, and WL. Flow cytometry and data analysis: ZS, QD, and JW. Drafting of manuscript and figures: ZS and QD. Critical revision of the manuscript: HZ. Statistical analysis: ZS, XW, and HC. Obtained funding: ZS and HZ. All authors contributed substantially to this manuscript and approved the final version of the manuscript.

## Funding

This work was funded by the Department of Science and Technology of Sichuan Province (Grant No. 2021YFS0173 and 2022YFS0315), the 1·3·5 project for disciplines of excellence–Clinical Research Incubation Project, West China Hospital, Sichuan University (Grant No. 21HXFH041), and the China Postdoctoral Science Foundation (Grant No. 2021M692295).

## Acknowledgments

The authors thank all the patients and healthy volunteers for their participation.

## Conflict of interest

The authors declare that the research was conducted in the absence of any commercial or financial relationships that could be construed as a potential conflict of interest.

## Publisher’s note

All claims expressed in this article are solely those of the authors and do not necessarily represent those of their affiliated organizations, or those of the publisher, the editors and the reviewers. Any product that may be evaluated in this article, or claim that may be made by its manufacturer, is not guaranteed or endorsed by the publisher.
