# Supplementary material for: Granzyme B in circulating CD8+ T cells as a biomarker of immunotherapy effectiveness and disability in neuromyelitis optica spectrum disorders
Source: Front Immunol. 2022 Nov 9;13:1027158. doi: 10.3389/fimmu.2022.1027158 (PMC9682179; doi:10.3389/fimmu.2022.1027158)
Supplement: Supplementary file 1 [file DataSheet_1.docx]

**Supplementary**

**
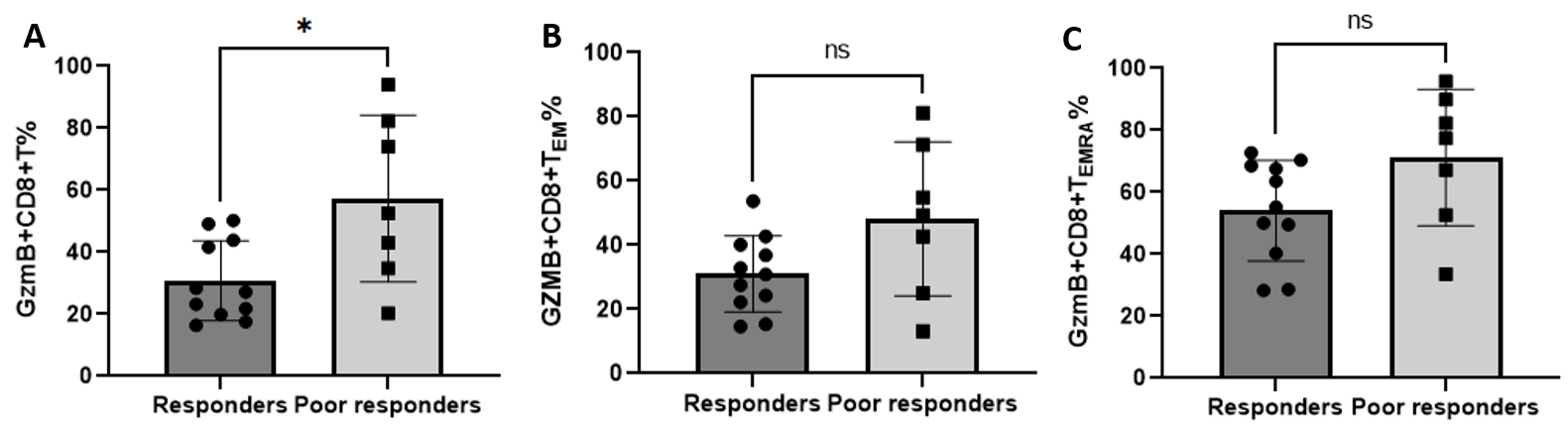
**

**Figure 1 GzmB-expressing CD8+ T cells between responders and poor responders to MMF.** Comparison of GzmB+CD8+ T% (A), GzmB+CD8+ T_EM_ (B), but not in GzmB+CD8+ T_EMRA_% (C) between responders to MMF (n=11) and poor responders to MMF (n=7). MMF, mycophenolate mofetil. *P < 0.05 and ns (not significant) by Mann–Whitney U-test.
